# Nutritional and Gastroenterological Monitoring of Patients With Celiac Disease During COVID-19 Pandemic: The Emerging Role of Telemedicine and Point-of-Care Gluten Detection Tests

**DOI:** 10.3389/fnut.2021.622514

**Published:** 2021-04-13

**Authors:** Andrea Costantino, Leda Roncoroni, Daniele Noviello, Nicoletta Nandi, Vincenza Lombardo, Alice Scricciolo, Lucia Scaramella, Maurizio Vecchi, Luca Elli

**Affiliations:** ^1^Gastroenterology and Endoscopy Unit, Fondazione IRCCS Ca' Granda Ospedale Maggiore Policlinico, Milan, Italy; ^2^Center for Prevention and Diagnosis of Celiac Disease, Fondazione IRCCS Ca' Granda Ospedale Maggiore Policlinico, Milan, Italy; ^3^Department of Biomedical, Surgical and Dental Sciences, Università degli Studi di Milano, Milan, Italy; ^4^Department of Pathophysiology and Transplantation, Università degli Studi di Milano, Milan, Italy

**Keywords:** telemedicine, telehealth, nutrition, celiac disease, televisits, gluten free diet, gluten detection test, COVID-19

## Abstract

**Background and Aims:** Since the beginning of the coronavirus disease 2019 (COVID-19) pandemic, telemedicine has been supporting many patients with chronic diseases worldwide. However, data on celiac disease (CeD) nutritional and gastroenterological remote monitoring are scanty. The aims of our study were to verify patients' trust in telemedicine and to evaluate the feasibility of telemedicine in nutritional monitoring.

**Material and Methods:** We used telemedicine in place of the scheduled but not provided follow-up visits during the first lockdown of the COVID-19 pandemic. Patients received a phone call, and televisits were conducted for CeD patients with mild or moderate symptoms and/or with blood alterations. The patient's adherence to the gluten-free diet (GFD) was evaluated according to the Celiac Dietary Adherence Test (CDAT). When gluten contamination was suspected, a point-of-care gluten detection test was prescribed. The patient's trust in telemedicine was assessed, through an adapted version of the Patient Trust Assessment Tool (PATAT) questionnaire, as the percentage of patients giving a score of at least 4 out of 5 on a Likert scale for three selected key statements: “I can trust televisit,” “I can trust that possible problems with the telemedicine service will be solved properly,” and “I feel at ease when working with this website.”

**Results:** One hundred and twelve CeD patients were phone called; among symptomatic patients, 39 out of the 42 scheduled (92.9%) televisits were performed. Among the 39 visits, 34 (87.2%) questionnaires were compiled. The patients included in the study obtained a CDAT score from 7 to 13 (11 ± 2). Gluten detection tests were prescribed to 11 patients, resulting positive in 2. Trust in the telemedicine service was achieved in 94.1, 88.2, and 97.1% for the three selected key statements of the PATAT questionnaire.

**Conclusion:** During the COVID-19 pandemic, telemedicine showed to be feasible and the majority of patients trusted the combined gastroenterological and nutritional televisits. Gluten detection tests demonstrated to be useful tools for the patient and for the caregiver to confirm adherence to the GFD remotely.

## Introduction

Telehealth is defined by the American Telemedicine Association (ATA) as “technology-enabled health and care management and delivery systems that extend capacity and access” (1). It includes not only health care delivery (often identified as telemedicine) in terms of disease diagnosis and treatment but also several other activities and services, such as prevention, education, and public health promotion (2). Telehealth is able to improve and increase access to health care, extending interaction with distantly located patients and enabling both patients and health care providers (HCPs) to have more flexible scheduling and greater efficiency in terms of cost and time (3).

Since the first reported cases of severe acute respiratory syndrome coronavirus 2 (SARS-CoV-2)-related pneumonia in China in December 2019, in just a few weeks, the virus had spread worldwide, leading to unforeseen consequences on every aspect of our daily working and social life as well as to radical changes in health care delivery. Italy was the first European country to experience the outbreak, and in particular, Lombardy and the areas of Bergamo, Lodi, and Milan were the very first “red areas” identified in the Country^1^. On March 9, 2020, the Italian government decided to place the whole country in a strict lockdown for almost 2 months. Moreover, in almost every Italian hospital, scheduled outpatient visits had been canceled, medical services were discontinued, and the medical staff were referred to perform other duties. In this scenario, telemedicine emerged as the ideal (and only) solution to overcome the impossibility of carrying out regular visits, allowing the continuation of patient assistance (4).

In the literature, only a few studies, before and during the coronavirus disease 2019 (COVID-19) pandemic, have evaluated the role and perception of telehealth and, in particular, televisits in gastroenterology and even fewer in celiac disease (CeD) (5, 6) and nutrition (7). These studies generally showed a positive result in favor of telemedicine, as televisits with patients in their homes for nutritional counseling appear to be appropriate since nutritional advice can be easily submitted to patients during such face-to-face consultation (6). Despite these studies and those performed in other fields mostly reporting positive outcomes in terms of satisfaction and cost-effectiveness, none of them investigate a perspective about patients' trust on telemedicine and televisits. Furthermore, the lack of data about the interactions among SARS-CoV-2 infection and CeD underlines the necessity to maintain a constant follow-up of patients (8).

CeD is a chronic autoimmune disease with a prevalence around 1%. The gluten-free diet (GFD) is the only effective treatment. However, a full adherence to the GFD is very difficult to achieve, and thus, patients should be followed up regularly for the assessment of symptoms and dietetic adherence. The newly introduced technologies for the detection of gluten in food and biological samples (urine and stool) can support CeD monitoring. Among them, urinary detection of gluten immunogenic peptides (GIP) is a self-administered point-of-care test with the aim to reveal unconscious gluten ingestions. It has been demonstrated that urinary GIP test is sensitive, specific, and effective to monitor GFD adherence (9, 10). Furthermore, questionnaires could be useful during CeD follow-up; the Celiac Dietary Adherence Test (CDAT) is a clinically relevant easily administered questionnaire which helps in the standardized evaluation of GFD adherence (11).

### Aims

The aims of our study were to verify CeD patients' trust in telemedicine and to evaluate the feasibility of telemedicine in CeD gastroenterological and nutritional monitoring.

## Methods

### Patients

During the general lockdown in Italy for the COVID-19 pandemic, we embraced telemedicine for our patients with CeD. From March 2020 to May 2020, phone calls were made in place of the previously scheduled but not carried out follow-up visits at our tertiary referral center “*Centre for Prevention and Diagnosis of Celiac Disease”* (Gastroenterology Unit, *Fondazione IRCCS Ca' Granda Ospedale Maggiore Policlinico*, Milan, Italy).

Televisits were proposed in addition to phone calls for every patient with symptoms (e.g., diarrhea, abdominal pain, weight loss) or in those with altered blood test results. In other cases, visits were postponed. Televisits were also offered to patients for nutritional counseling concerning CeD and GFD.

Video calling solutions from *Google* (*Hangouts* or *Meet*) or *Microsoft Teams* were used according to each patient's preference. Patients who did not have internet connection or were unable to use a smartphone device were necessarily excluded *a priori*.

With the term telemedicine, we intend phone calls, televisits, and remote point-of-care diagnostic test for gluten detection and GFD monitoring.

### Gluten-Free Diet Adherence

The patient's adherence to the GFD was evaluated according to the CDAT. The CDAT is a clinically relevant, easily administrated seven-item instrument which allows the standardized evaluation of GFD adherence. It is a sensitive tool developed using standard psychometric techniques. CDAT is based on a score ranging from 7 to 35 for seven questions, each on a five-point scale, with higher scores denoting worse GFD adherence (11).

In case of uncertain gluten contamination or presence of intestinal/extraintestinal symptoms, it was suggested to patients to self-verify the dosage of gluten urinary peptides following the manufacturer's instruction (9, 10). Gluten detection tests use G12 monoclonal antibody (MoAb) able to detect GIP in urine by the immunochromatographic technique. The positive result indicates that gluten intake was detected within the last 24–48 h (12).

### Trust in Telemedicine

Patients' trust in televisits was assessed through an adapted version of the Patient Trust Assessment Tool (PATAT) questionnaire (Table 1). The questionnaire investigated five trust areas: care organization, care professionals, treatment, technology, and telemedicine services (13). It was translated into Italian and formulated online on the *EUSurvey* platform by our center. After the televisit, each patient received an email containing the questionnaire URL and provided their informed consent before compiling the anonymous questionnaire. The questionnaire was formulated through the *EUSurvey* platform, which is widely used for clinical research questionnaires in Europe.

**Table 1:** Clinical and demographic characteristics of celiac patients who accepted the televisit.

	**CeD (*n* = 39)**
Age, years, median (range)	42.0 (20–73)
Female, *n* (%)	34 (87.2%)
Age at diagnosis (years)	31.0 (2–61)
Disease duration (years)	11.0
Refractory CeD	1 (2.6%)
Familiarity for CeD, *n* (%)	11 (28.2%)
Comorbidities, *n* (%)	25 (64.1%)

*CeD, celiac disease*.

Patients' trust in telemedicine was expressed as a percentage of patients >75% giving a score of at least 4 out of 5 on a Likert scale for three selected key statements: “I can trust televisit” (5.1), “I can trust that possible problems with the telemedicine service will be solved properly” (5.2), and “I feel at ease when working with this website” (5.4).

This study was approved by our local ethics committee (number 550/2020).

### Statistical Analysis

The demographic data were described as median (range) or unless otherwise indicated. The patients' trust was expressed as total number and percentage. The continuous variables were compared using independent Student's *t*-test. Fisher's exact test was used to evaluate the distribution of categorical variables. The statistical analysis was performed by SPSS software ver. 26.0 (IBM, Armonk, NY, US). Following the previously reported data on patients' trust in telemedicine (14, 15)/*post hoc* power analysis was performed. A power (β – 1) >80% with a two-sided 5% significance level was considered acceptable (16) (G^*^Power package ver. 3.1.9.4, University of Dusseldorf, http://www.gpower.hhu.de.).

## Results

During the March 2020 lockdown, we phone called 112 CeD patients scheduled to undergo routine gastroenterological and nutritional visits.

**Figure 1:**
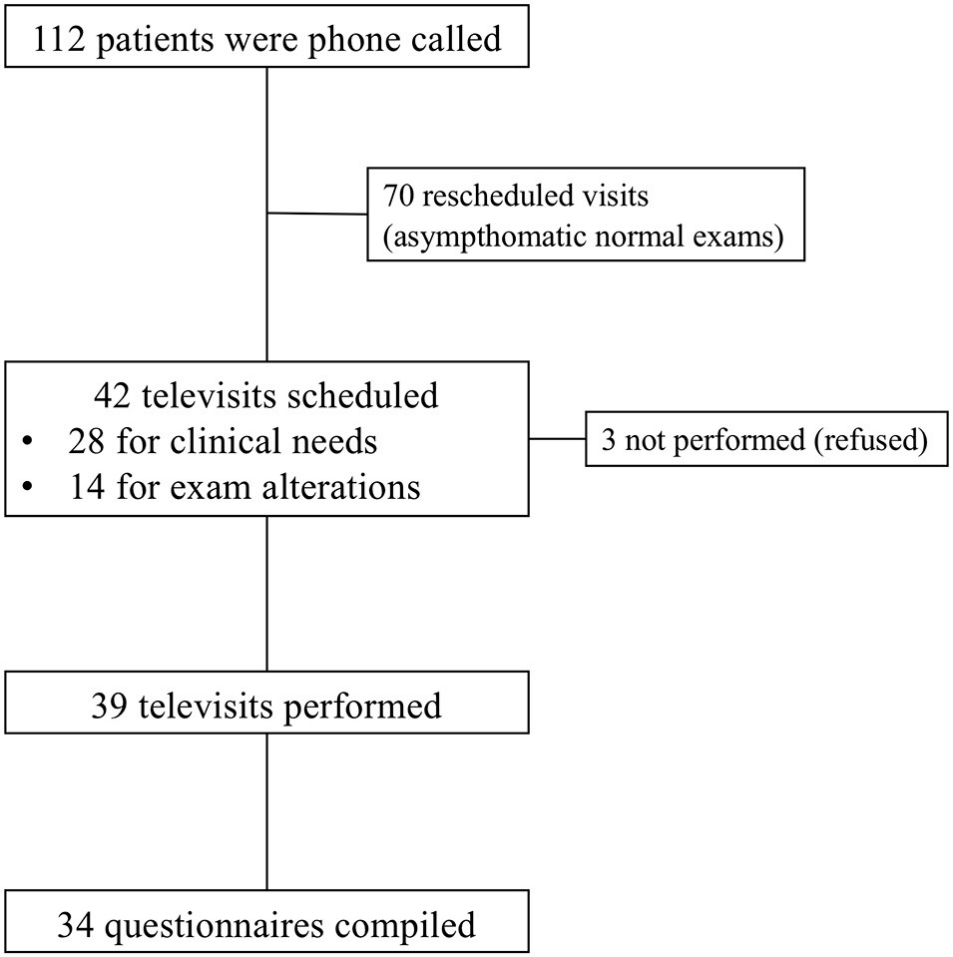
Flowchart of the study.

Thus, we scheduled 42 televisits (37.5%) for every patient with symptoms or altered blood test results; among them, 39 (92.9%) televisits were successfully performed. Three patients refused to perform the televisit and preferred the in-person visit (Figure 1).

The baseline characteristics of the patients who accepted televisits are shown in Table 1. No demographic differences were observed between the group undergoing televisit and the postponed patients (data not shown).

All included patients reported a very good adherence to the GFD, expressed as CDAT score ranging from 7 to 13 (11 ± 2). Eleven patients reported the presence of symptoms despite referring adherence to GFD but with a suspect of possible contaminations. To those patients, we prescribed gluten detection tests which resulted positive in two cases (18.2%). Thirty-four (87.2%) questionnaires about trust in telemedicine were compiled after the nutritional and gastroenterological televisits (Figure 1, Table 2).

**Table 2:** The patient trust assessment tool (PATAT) as responded by 34 CeD patients.

		**Percentage of patients giving a score ≤3**	**Percentage of patients giving a score ≥4**
**1**	**Trust in the care organization**		
1.1	The Celiac Centre Polyclinic of Milan has a good reputation.	0%	100%
1.2	At the Celiac Centre Polyclinic of Milan, they handle my personal information carefully.	2.9%	97.1%
1.3	At the Celiac Centre Polyclinic of Milan, they take action when something goes wrong.	2.9%	97.1%
1.4	At the Celiac Centre Polyclinic of Milan, I feel at ease.	0.0%	100%
1.5	At the Celiac Centre Polyclinic of Milan, they take my specific needs into account.	2.9%	97.1%
**2**	**Trust in care professional**		
2.1	I trust my doctor's judgement about my medical care.	0%	100%
2.2	My doctor provides me with all the information on all potential medical options.	0%	100%
2.3	My doctor keeps all my medical information private.	2.9%	97.1%
2.4	I always follow my doctor's advice.	5.9%	94.1%
2.5	My doctor does not do everything they should about my medical care.	85.3%	14.8%
**3**	**Trust in treatment**		
3.1	The treatment I receive is effective.	8.8%	91.2%
3.2	It is clear to me what the treatment I receive entails.	2.9%	97.1%
3.3	Together, my doctor and I made the choice for this treatment.	17.4%	73.5%
3.4	The treatment I receive is not helping me enough.	97.1%	2.9%
3.5	It has been explained well to me what my treatment entails.	2.9%	97.1%
**4**	**Trust in technology**		
4.1	When I use Google/Microsoft video service, I am in control.	5.9%	94.1%
4.2	Everything that I do on Google/Microsoft video service remains private.	8.8%	91.2%
4.3	The personal information that is stored at Google/Microsoft will not get lost.	23.5%	76.5
4.4	Google/Microsoft video service is easy to use.	5.9%	94.1%
4.5	Legal policy and technological safeguards make Google/Microsoft video service a safe environment.	8.8%	91.2%
**5**	**Trust in telemedicine service**		
5.1	I can trust this telemedicine service.	5.9%	94.1%
5.2	I can trust that possible problems with this telemedicine service will be solved properly.	11.8%	88.2%
5.3	I can trust this service less than other online services.	82.3%	17.7%
5.4	I feel at ease when working with Google/Microsoft video service.	2.9%	97.1%
5.5	I do not like to enter my personal data on Google/Microsoft.	79.4%	21.6%

*Trust in telemedicine was expressed as a percentage of patients >75% giving a score of at least 4 out of 5 on a Likert scale for three selected key statements: “I can trust televisit” (5.1), “I can trust that possible problems with the telemedicine service will be solved properly” (5.2), and “I feel at ease when working with this website” (5.4)*.

**Figure 2:**
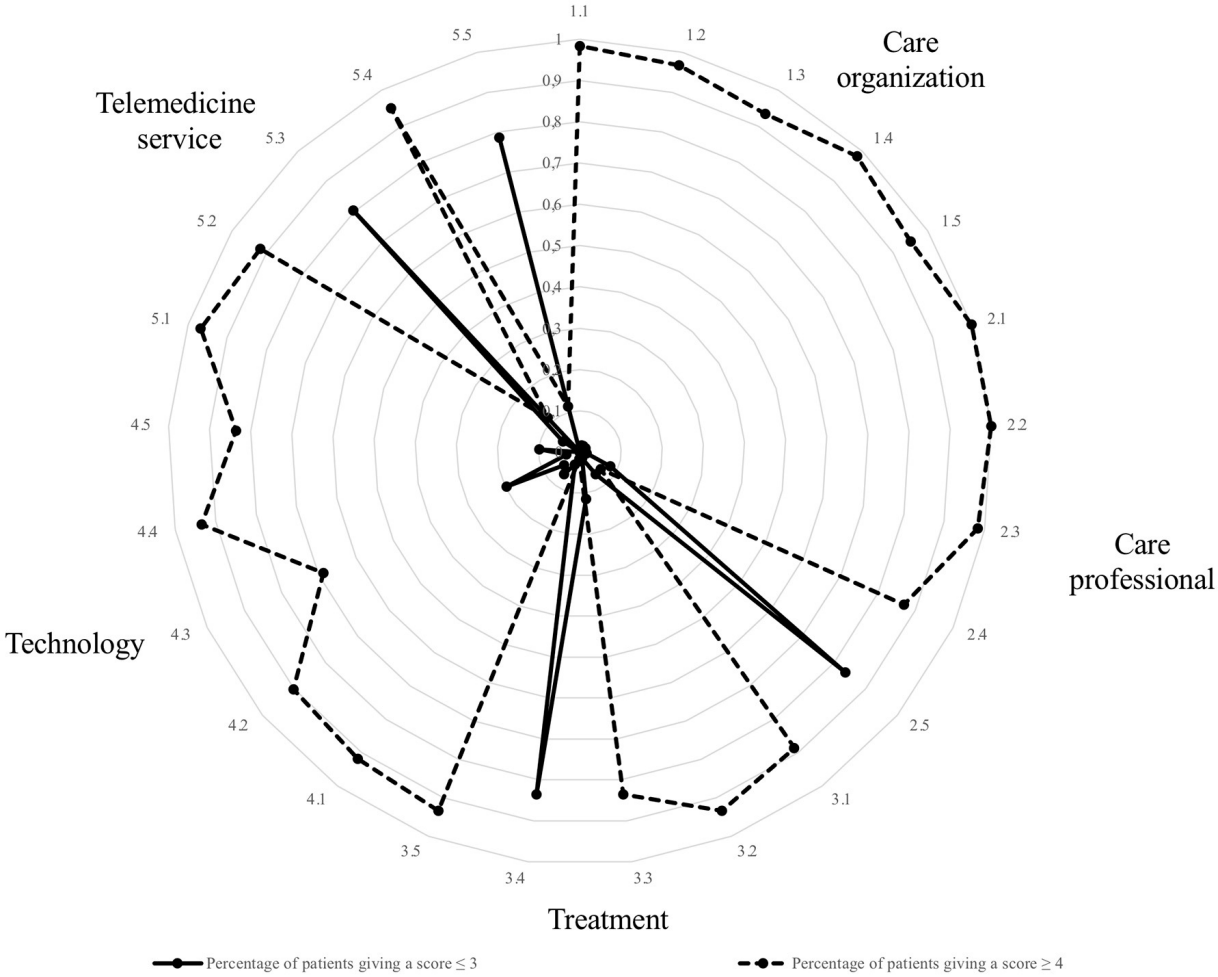
Radar chart of the trust scores from celiac patients assessed through an adapted version of the Patient Trust Assessment Tool (PATAT) questionnaire. Five trust areas were investigated: care organization (1.1–1.5), care professionals (2.1–2.5), treatment (3.1–3.5), technology (4.1–4.5), and telemedicine services (5.1–5.5). The dotted line refers to the patients giving a score ≥4 (out of 5) on a Likert scale. The continuous line refers to the patients giving a score ≤ 3. Statements 2.5, 3.4, 5.3, and 5.5 were negative. Regarding trust in telemedicine services, items 5.1, 5.2, and 5.4 received a score of least 4 in 95, 90, and 84% of the cases, respectively.

Regarding trust in the telemedicine service, items 5.1, 5.2, and 5.4 received a score of least 4 in 94.1, 88.2, and 97.1% of the patients, respectively (Figure 2). Findings from the PATAT questionnaire are reported in Table 2. According to the previously reported patients' trust in telemedicine, ranging from 50 to 60%, the estimated power was >80% in case of comparison with the trust showed by the analyzed cohort of CeD subjects.

The questionnaire results showed that, during the COVID-19 pandemic, CeD patients who were followed at our center agreed to receive a televisit in spite of the traditional in-person visit and they trusted televisits.

## Discussion

Telemedicine has been often recognized as a valuable tool with great potential. Nevertheless, its role is still marginal in daily clinical practice, as communication with patients occurs largely *via* emails and texts, even though patients often find these forms of communication unsatisfactory due to the offline interaction and the delay in response (17). It should also be considered that up until December 2020 (15), there were no laws or regulations by the Italian National Health System officially directing or recognizing telemedicine as a tool to perform and deliver health care.

Recently, a study conducted by the Universities of Padua and Salerno among adult CeD patients aimed to assess their perception of COVID-19 effects. When asked about their opinion on remote telemedicine visits, most of them responded that they were “happy with it” (86%) and part of them explicitly requested it (~17%) (6).

The experience gathered during the COVID-19 pandemic will probably further reinforce pre-COVID data on the effectiveness and good performance of telemedicine and push for a more widespread adoption in regular clinical practice even beyond the emergency pandemic. Our study demonstrated the good feasibility and the positive attitude of CeD patients toward telemedicine. This positive trust rate in televisits among CeD patients is a fundamental prerequisite for having confidence in proposing this approach and achieving a successful implementation. The high percentage of patients observed in the study is certainly due to the high number of patients who trust the *Center for Prevention and Diagnosis of Celiac Disease* and its doctors. It is likely that such trust can instill confidence in the proposed telemedicine service even if trust in the technology itself does not have such high values and that the pandemic has helped increase the usual trust in the performance performed away from a doctor's office. A possible limitation of our study is that the vast majority of the patients who had the televisits due to CeD were women, and males are less inclined to follow-up and carry out with medical appointments. However, similar results were observed in a previous study on patients with inflammatory bowel diseases and there was a male sex predominance (18).

To date, thanks to advanced communication technologies, most commonly computers and mobile phones, HCPs can interact long distance with patients *via* synchronous modalities (mainly live videoconferencing allowing for interactive consultation and immediate interventions) and asynchronous modalities (2). However, the spread of telemedicine may be limited by patients who are not familiar with digital technologies. In our experience, in-person visits were maintained for those who did not have internet access and/or technological devices or were not capable of using them. Alternatively, a patient's caregiver could help for this portion of patients eventually excluded from remote assistance.

Nevertheless, our study may change our propensity leading HCPs to more consistently adopt telemedicine and expand its use beyond the traditional ideal setting (e.g., young patient, digital workers) and beyond the emergency pandemic context.

Considering that strict adherence to a GFD is the only therapy in patients with CeD (excluding patients with refractory celiac disease), remote monitoring could play an important role. Gastroenterological and nutritionist televisits or phone triage could be performed using validated easy-to-administer questionnaires, which also seem to be an optimal tool even in an emergency setting such as the COVID-19 pandemic. Furthermore, in selected cases, the use of point-of-care and self-administered tests to monitor GFD (such as urinary GIP) could give an important support. A proportional fraction of GIP absorbed reaches the circulation and is excreted in the urine, allowing for a better evaluation of patients' adherence to GFD compared to serology (which has low specificity and sensitivity in determining both adherence to the gluten-free diet and healing of the intestinal mucosa) and to frequent repetition of biopsies (19). It has been demonstrated that they are a valuable aid for celiac patients to monitor their GFD, improving adherence and checking for accidental gluten ingestion (9, 12). Moreover, these point-of-care tests are easy-to-use, low-cost, reliable, and accurate tools to verify possible gluten contamination even during remote televisits.

Considering telemedicine innovation and its potential risks (privacy issues or possible medical errors), only few televisits have been performed in our study. Therefore, the expression of patients' trust in televisit was evaluated in a small group, and it is a good representative of those who had the televisit. Nevertheless, since the aim of our study was not to analyze what influenced the level of trust among different groups, it may represent well the general trust in telemedicine in a larger CeD population.

In the near future, it is likely that telemedicine is going to be used for visiting patients who are asymptomatic and with a mild disease as well as for maintaining normal visits for patients with mild or severe disease or with symptoms. In addition, telemedicine could be used as a triage visit in order to select patients who request further exams or in-person visits as well as those who do not.

Whether this trust in telemedicine will last when the COVID-19 pandemic will eventually be over and how telemedicine should be better deployed will surely be further analyzed.

## Conclusion

During the COVID-19 pandemic, telemedicine has been a powerful and convenient tool for patients with CeD to gain access to remote assistance; at the same time, it has potentially contained SARS-CoV-2 spreading among patients and HCPs. We had the possibility to perform a televisit in more than 90% of symptomatic patients, and the majority of these patients trusted the combined gastroenterological and nutritional televisits. Gluten detection tests demonstrated to be useful tools for the patient and for the caregiver to confirm adherence to the GFD or accidental gluten contamination remotely.

## Data Availability Statement

The raw data supporting the conclusions of this article will be made available by the authors, on reasonable request to the corresponding author.

## Ethics Statement

This study, involving human participants, was reviewed and approved by Ethics Committee Milano Area 2, n. 550/2020. The patients/participants provided their informed consent to participate in this study.

## Author Contributions

AC and DN designed the study, carried out the acquisition, analysis and interpretation of data. AC, NN, LR, and DN wrote the manuscript. VL, LS, and AS gave material support for video-consultations. LE and MV supervised the study and critically reviewed the manuscript. All authors contributed to the article and approved the submitted version.

## Conflict of Interest

The authors declare that the research was conducted in the absence of any commercial or financial relationships that could be construed as a potential conflict of interest.

## Footnotes

^1^http://www.salute.gov.it/portale/nuovocoronavirus/dettaglioNotizieNuovoCoronavirus.jsp?lingua=italiano&menu=notizie&p=dalministero&id=4370 (accessed September 14, 2020).

## References

[1] American Telemedicine Association. American Telemedicine Association (ATA), Telehealth Basic. Available online at: https://www.americantelemed.org/resource/why-telemedicine/ (accessed January 23, 2021).

[2] Wilson LS Maeder AJ. Recent directions in telemedicine: review of trends in research and practice. Healthc Inform Res. (2015) 21:213–22. 10.4258/hir.2015.21.4.213 26618026 PMC4659877

[3] Dorsey ER Topol EJ. State of telehealth. N Engl J Med. (2016) 375:154–61. 10.1056/NEJMra1601705 27410924

[4] Elli L Barisani D Vaira V Bardella MT Topa M Vecchi M. How to manage celiac disease and gluten-free diet during the COVID-19 era: proposals from a tertiary referral center in a high-incidence scenario. BMC Gastroenterol. (2020) 20:387. 10.1186/s12876-020-01524-4 33213379 PMC7675390

[5] Vriezinga S Borghorst A van den Akker-van Marle E Benninga M George E Hendriks D. E-Healthcare for celiac disease-a multicenter randomized controlled trial. J Pediatr. (2018) 195:154–60.e7. 10.1016/j.jpeds.2017.10.027 29275927

[6] Siniscalchi M Zingone F Savarino EV D'Odorico A Ciacci C. COVID-19 pandemic perception in adults with celiac disease: an impulse to implement the use of telemedicine. Dig Liver Dis. (2020) 52:1071–5. 10.1016/j.dld.2020.05.014 32425731 PMC7229921

[7] Rollo ME Hutchesson MJ Burrows TL Krukowski RA Harvey JR Hoggle LB. Video consultations and virtual nutrition care for weight management. J Acad Nutr Diet. (2015) 115:1213–25. 10.1016/j.jand.2015.03.016 25986214

[8] Elli L Scaramella L Lombardo V Scricciolo A Doneda L Roncoroni L. Refractory celiac disease and COVID-19 outbreak: findings from a high incidence scenario in Northern Italy. Clin Res Hepatol Gastroenterol. (2020) 44:e115–20. 10.1016/j.clinre.2020.07.026 32893177 PMC7442891

[9] Moreno ML Cebolla Á Muñoz-Suano A Carrillo-Carrion C Comino I Pizarro Á. Detection of gluten immunogenic peptides in the urine of patients with coeliac disease reveals transgressions in the gluten-free diet and incomplete mucosal healing. Gut. (2017) 66:250–7. 10.1136/gutjnl-2015-310148 26608460 PMC5284479

[10] Costa AF Sugai E Temprano MP Niveloni SI Vázquez H Moreno ML. Gluten immunogenic peptide excretion detects dietary transgressions in treated celiac disease patients. World J Gastroenterol. (2019) 25:1409–20. 10.3748/wjg.v25.i11.1409 30918433 PMC6429344

[11] Leffler DA Dennis M Edwards George JB Jamma S Magge S Cook EF. A simple validated gluten-free diet adherence survey for adults with celiac disease. Clin Gastroenterol Hepatol. (2009) 7:530–6, 536.e1-2. 10.1016/j.cgh.2008.12.032 19268725

[12] Soler M Estevez MC Moreno Mde L Cebolla A Lechuga LM. Label-free SPR detection of gluten peptides in urine for non-invasive celiac disease follow-up. Biosens Bioelectron. (2016) 79:158–64. 10.1016/j.bios.2015.11.097 26703993

[13] Velsen LV Tabak M Hermens H. Measuring patient trust in telemedicine services: development of a survey instrument and its validation for an anticoagulation web-service. Int J Med Inform. (2017) 97:52–8. 10.1016/j.ijmedinf.2016.09.009 27919395

[14] Russo L Campagna I Ferretti B Agricola E Pandolfi E Carloni E. What drives attitude towards telemedicine among families of pediatric patients? A survey. BMC Pediatr. (2017) 17:21. 10.1186/s12887-016-0756-x 28095894 PMC5240275

[15] Welch BM Harvey J O'Connell NS McElligott JT. Patient preferences for direct-to-consumer telemedicine services: a nationwide survey. BMC Health Serv Res. (2017) 17:784. 10.1186/s12913-017-2744-8 29183372 PMC5704580

[16] Faul F Erdfelder E Lang AG Buchner A. G^*^Power 3: a flexible statistical power analysis program for the social, behavioral, and biomedical sciences. Behav Res Methods. (2007) 39:175–91. 10.3758/BF03193146 17695343

[17] Daschle T Dorsey ER. The return of the house call. Ann Intern Med. (2015) 162:587–8. 10.7326/M14-2769 25894028

[18] Costantino A Noviello D Mazza S Berté R Caprioli F Vecchi M. Trust in telemedicine from IBD outpatients during the COVID-19 pandemic. Dig Liver Dis. (2021) 53:291–4. 10.1016/j.dld.2020.10.035. 33187917 PMC7644233

[19] Elli L Bascuñán K di Lernia L Bardella MT Doneda L Soldati L. Safety of occasional ingestion of gluten in patients with celiac disease: a real-life study. BMC Med. (2020) 18:42. 10.1186/s12916-020-1511-6 32172690 PMC7075003

